# Enhancing caries-affected dentin bonding with a mussel-inspired primer

**DOI:** 10.3389/fbioe.2025.1574562

**Published:** 2025-04-28

**Authors:** Yuntong Hu, Yi He, Dingjie Wang, Yingjing Wei, Xiaodong Xing, Yuhong Xiao

**Affiliations:** ^1^ Department of Oral Surgery, 920th Hospital of Joint Logistics Support Force, PLA, Teaching Hospital of Kunming Medical University, Kunming, China; ^2^ Yunnan Key Laboratory of Stomatology and Department of Dental Research, Kunming Medical University, Kunming, China; ^3^ Department of Stomatology, The First Hospital of Kunming, Teaching Hospital of Kunming Medical University, Kunming, China; ^4^ College of Chemical Engineering, Nanjing University of Science and Technology, Nanjing, China

**Keywords:** dental adhesives, dental diseases, mussel, cross-linking reagents, collagen, antibacterial

## Abstract

**Introduction:**

Dental caries is the most common oral disease. In caries-affected dentin (CAD), excessive mineral loss, extensive collagen exposure and collapse, increased enzyme activity, and bacterial residues result in significantly lower resin bonding strength and durability compared to sound dentin (SD). Currently, there are no effective clinical strategies to enhance CAD bonding. Inspired by the excellent wet adhesion capability and collagen affinity of marine mussels, this study aimed to evaluate a mussel-inspired polymerizable monomer (catechol–Lys–methacrylate [CLM]) as a primer to improve CAD bonding performance.

**Methods:**

The interactions between CLM and collagen were analyzed *via* Fourier-transform infrared spectroscopy (FTIR) and nuclear magnetic resonance (NMR). Microtensile bond strength, nanoleakage, *in-situ* zymography, and sodium dodecyl sulfate-polyacrylamide gel electrophoresis (SDS-PAGE) were used to assess the bond strength and interface stability. Furthermore, the antibacterial properties of CLM were evaluated using colony-forming units counts, live/dead bacterial staining, and bacterial morphology observation.

**Results:**

FTIR and NMR results showed that CLM was successfully grafted onto CAD collagen through its catechol groups, facilitating subsequent chemical bonding with resin. CLM increased the immediate CAD bond strength by approximately 30% and reduced immediate nanoleakage by approximately 24%, maintaining effectiveness after aging. Moreover, collagen chemical modification by CLM promoted collagen crosslinking, inhibited endogenous enzymatic activity, and conferred antibacterial properties, further enhancing bonding interface stability.

**Discussion:**

In summary, this study reports the application of a mussel-inspired monomer, CLM, in CAD bonding. During the wet bonding process, CLM not only improves collagen stability but also serves as a molecular bridge between inorganic resin and organic collagen, thereby enhancing both immediate and aged bonding performance. These findings showing promising clinical application potential.

## 1 Introduction

Dental caries, the most prevalent oral disease, affects over 2.5 billion people worldwide (Jain et al., 2024). The primary clinical approach to caries treatment involves mechanically removing infected dental tissue and restoring the tooth’s shape and function with resin materials (Cadenaro et al., 2023). However, according to the principles of modern minimally invasive dentistry, caries treatment should prioritize pulp preservation. This approach selectively removes carious tissue down to the softened dentin at the cavity floor while retaining part of the caries-affected dentin (CAD) for restorative treatment (Schwendicke et al., 2016). However, the bonding strength and durability of CAD are significantly lower than those of sound dentin (SD), making CAD bonding a persistent challenge in dental adhesion (Almahdy et al., 2015).

Poor CAD bonding performance mainly results from the collapse of the collagen network and excessive hydroxyapatite loss, hindering adhesive resin penetration and significantly reducing the bond strength (Donmez et al., 2019; Suppa et al., 2006). Increased enzyme activity in CAD leads to easier demineralized collagen degradation (Fialho et al., 2019; Mazzoni et al., 2015). Additionally, residual cariogenic bacteria within CAD thrive in the anaerobic environment after filling, increasing the risk of secondary caries (Mohammed et al., 2014). Previous research on improving CAD bonding mainly focused on collagen crosslinking or enzyme inhibition (Davila-Sanchez et al., 2020; Hass et al., 2021; Macedo et al., 2009); however, enzyme inhibition alone does not significantly enhance the CAD bond strength. Current commercial self-etch adhesive systems primarily bind chemically to hydroxyapatite; however, extensive hydroxyapatite loss in CAD diminishes these chemical bonding effects (Isolan et al., 2018). Despite the presence of abundant collagen in CAD, existing adhesives only mechanically interlock with collagen, and there are no stable chemical bonds (Mazzoni et al., 2015). Therefore, developing novel monomers capable of chemical bonding with collagen could significantly improve CAD bonding.

Marine mussels exhibit strong adhesion in wet environments due to adhesive proteins secreted by mussel foot glands. 3,4-dihydroxyphenylalanine (DOPA), the critical adhesive component (Li et al., 2020), displaces interfacial water and acts as an ideal bridging molecule through hydrogen, covalent, and electrostatic bonds between inorganic and organic phases (Xu et al., 2018; 2023). Dental adhesives face similar challenges to mussels, requiring durable adhesion in moist environments. Artificial DOPA derivatives like dopamine methacrylamide (DMA) have improved resin-dentin interfaces by inhibiting collagenase activity and promoting crosslinking (Li et al., 2021).

**FIGURE 1:**
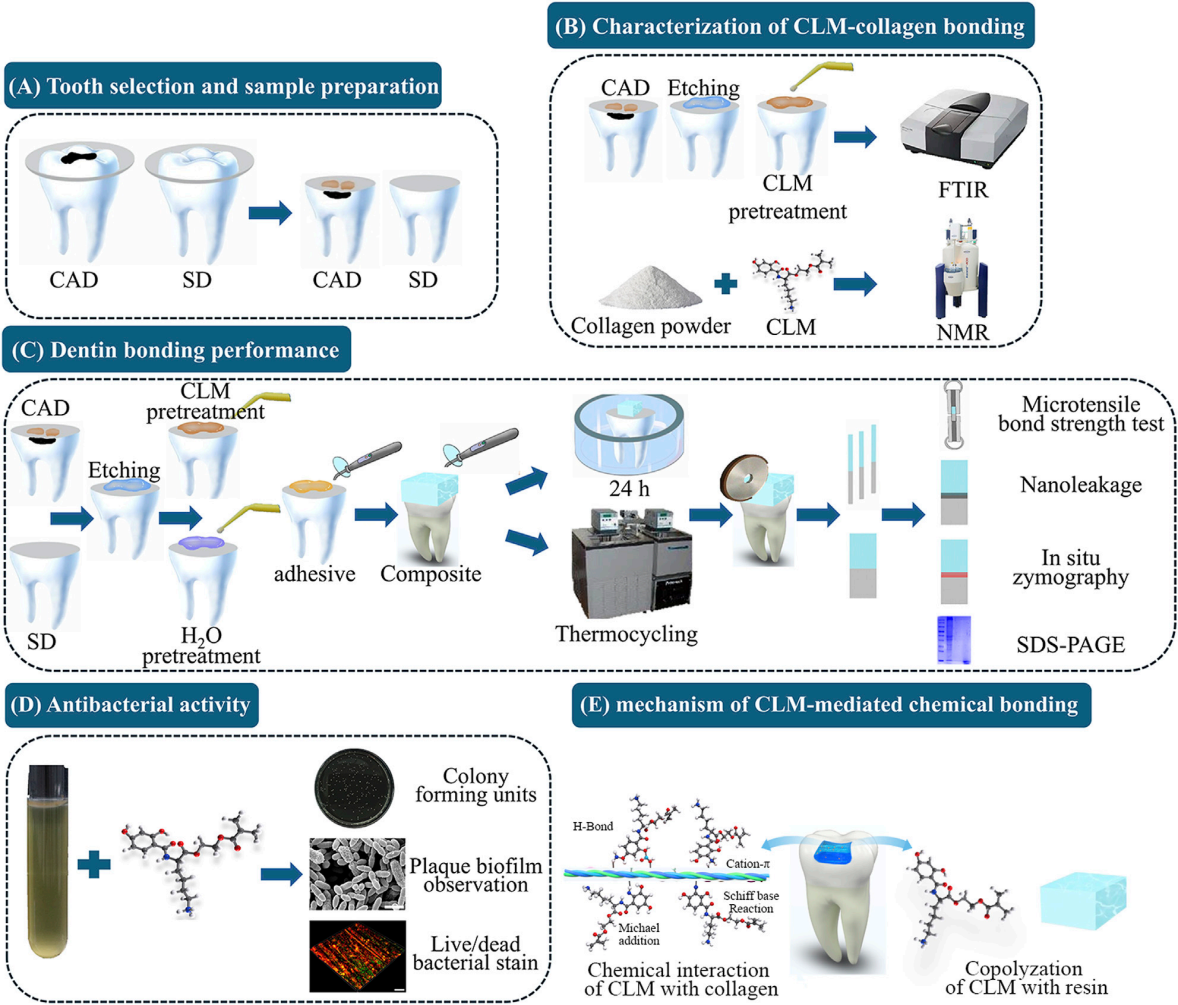
Schematic illustration of the experimental procedure and the mechanism of catechol-lysine methacrylate (CLM)-mediated chemical bonding. **(A)** Tooth selection and sample preparation: Intact and carious third molars were selected to represent sound dentin (SD) and caries-affected dentin (CAD), respectively. Dentin surfaces were exposed by cutting at one-third of the occlusal dentin thickness. **(B)** Characterization of CLM-collagen bonding: Fourier transform infrared spectroscopy (FTIR) and nuclear magnetic resonance (NMR) spectroscopy were used to characterize the interaction between CLM and collagen. **(C)** Dentin bonding performance: Microtensile bond strength (μTBS) tests, nanoleakage evaluation, *in situ* zymography, and sodium dodecyl sulfate–polyacrylamide gel electrophoresis (SDS-PAGE) were used to evaluate the effect of the CLM primer on CAD bonding performance. **(D)** Antibacterial activity: Colony-forming unit (CFU) assays, plaque biofilm observations, and live/dead bacterial staining were used to evaluate the antibacterial activity of CLM. **(E)** The mechanism of CLM-mediated chemical bonding.

However, derivatives solely mimicking catechol structures differ from natural mussel proteins. Lysine (Lys) residues adjacent to catechol in mussel proteins synergistically achieve underwater adhesion (Maier et al., 2015; Rapp et al., 2016). DMA, mimicking only the catechol structure, overlooks this synergy, limiting effectiveness. To address these limitations, our group synthesized a novel monomer—catechol-lysine methacrylate (CLM)—which combines lysine’s water displacement capability with catechol’s chemical bonding to collagen (Hu et al., 2023). CLM also contains polymerizable methacrylate groups, enabling it to bridge collagen and resin effectively, potentially improving CAD bonding. This study aimed to evaluate CLM primer effectiveness in enhancing CAD bonding (Figure 1). We tested two hypotheses: The experimental CLM primer would not affect the following: 1) immediate and aged resin-CAD bond strength and nanoleakage; 2) endogenous enzymatic activity at the resin-CAD bonding interface.

## 2 Materials and methods

### 2.1 Synthesis of CLM and preparation of the primer

**FIGURE 2:**
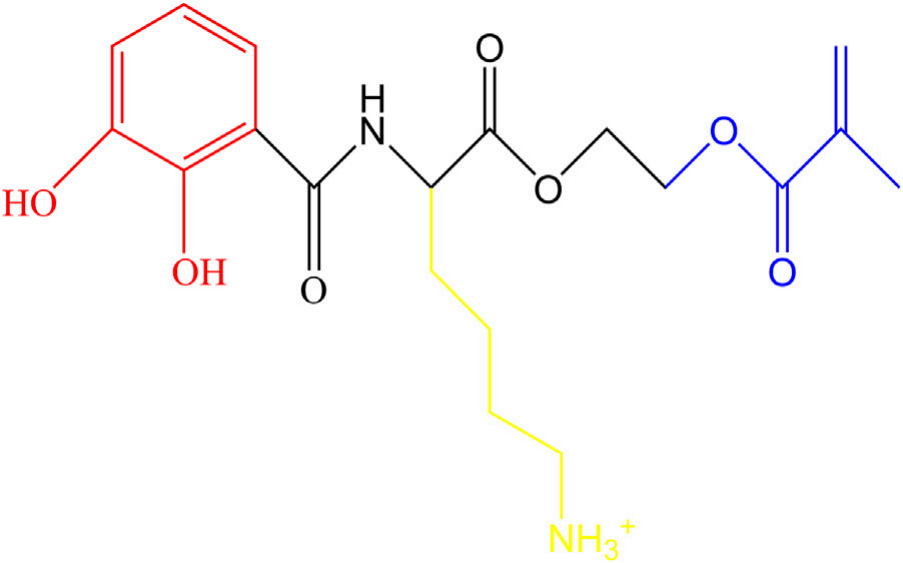
Molecular structure of CLM. Catechol groups are highlighted in red, lysine residues in yellow, and methacrylate groups in blue.

CLM was synthesized using Fmoc-L-Lys (Boc), 2,3-Dihydroxybenzoic acid, and 2-Hydroxyethyl methacrylate as raw materials (Figure 2) following our previously reported method. To prepare the CLM primer, 5 mg of CLM was dissolved in 1 mL of deionized water, yielding a 5 mg/mL solution (Hu et al., 2023).

### 2.2 Sample preparation

This study was approved by the Ethics Committee of the 920th Hospital of the Joint Logistics Support Force of the Chinese People’s Liberation Army (2019-022-01). Freshly extracted intact or carious third molars were collected with informed consent from patients and stored in 5% chloramine T solution at 4°C, to be used within 1 month.

X-ray films were used to identify extracted teeth with caries located within the occlusal one-third dentin. The enamel was removed parallel to the occlusal plane using a slow-speed saw (SYJ-150, Shenyang Kejing, China) under running water, exposing the CAD. The CAD surface was polished with 600-grit silicon carbide paper to obtain a standardized smear layer. SD specimens were prepared similarly.

For bonding procedures, dentin surfaces in each group were etched for 15 s with 35% phosphoric acid gel (Bisco Inc., Schaumburg, IL, United States), rinsed for 30 s with deionized water, and excess water was gently removed using filter paper. The wet bonding technique was employed, with moisture visible but without pooling. The experimental group was treated with CLM primer for 60 s, while the control group received deionized water for 60 s. Excess liquid was again removed with filter paper. A dental adhesive (Single Bond 2, 3M, United States) was then applied for 20 s, thinned gently for 5 s with mild airflow to remove solvents, and light-cured for 20 s using an LED curing unit at a distance of approximately 1 mm (1,470 mW/cm^2^; EliparTM DeepCure-S, 3M, United States). Composite build-ups (Filtek Z250, 3M ESPE, United States) were placed incrementally at 1.0 mm thickness for each layer, using circumferential matrix bands (Palofent 360, Dentsply, United States). Bonded specimens from each group were divided into two subgroups—immediate (stored in distilled water for 24 h) and aged (subjected to thermocycling at 5°C for 1 min and 55°C for 1 min, for a total of 10,000 cycles).

### 2.3 Characterization of the CLM-collagen interaction

#### 2.3.1 Fourier-transform infrared spectroscopy (FTIR)

CAD slices (approximately 1 mm thickness, n = 6) were prepared and etched with 35% phosphoric acid for 15 s, rinsed with deionized water for 30 s, and excess surface water was removed. Five specimens were treated with CLM primer for 60 s, while one specimen remained untreated as a control. Excess fluid was removed, and FTIR spectra were recorded using an attenuated total reflectance Fourier-transform infrared spectrometer (ATR-FTIR; Shimadzu FTIR-8400S, Japan). Spectra were collected within the range of 500–4,000 cm^−1^ at 4 cm^−1^ resolution for 24 scans to assess interactions between CLM and demineralized CAD (Tang et al., 2023).

#### 2.3.2 Nuclear magnetic resonance (NMR)

Type I collagen powder (CLP-01, Koken Co. Ltd., Japan) was dissolved in a buffer solution containing 50 mM d_4_-acetic acid, 150 mM NaCl, 5 mM CaCl_2_, and 0.02% NaN_3_ (pD adjusted to 4.0 with NaOD). The collagen solution was diluted fourfold with buffer to reduce viscosity. The CLM monomer was added to the diluted collagen solution (final concentration 5 mg/mL). NMR experiments were performed using a 600 MHz spectrometer equipped with a cryogenic probe (Bruker BioSpin Corporation, Billerica, United States). Spectra were acquired with a scan rate of 220 Hz, a relaxation delay of 1 s, and a sampling number of 4,096 × 256 to characterize the interactions between CLM and collagen (Hiraishi et al., 2013).

### 2.4 Dentin bonding performance

#### 2.4.1 Microtensile bond strength (μTBS)

A total of 30 M were randomly divided into groups (n = 5 per group). Resin-dentin beams (1 mm^2^ cross-sectional area, two beams per tooth, n = 10 beams/group) were sectioned using a slow-speed saw. μTBS testing was performed using a microtensile testing machine (EZ-TEST 500 N, Shimadzu Co., Japan) at a crosshead speed of 1 mm/min. The average μTBS values from four measurements per tooth were calculated (Tezvergil-Mutluay et al., 2010).

#### 2.4.2 Nanoleakage evaluation

Bonded specimens from SD and CAD groups (immediate and aged; three teeth per subgroup, a total six specimens per group) were sectioned perpendicular to the bonding interface into approximately 1 mm thick slabs. The slabs were coated with hydrophobic nail polish except within 1 mm around the bonding interface, then immersed in 50 wt% ammoniacal silver nitrate solution in the dark for 24 h. After developing for 8 h and fixing for another 8 h, samples were wet-polished (600, 1,200, 2000-grit SiC paper), ultrasonically cleaned for 5 min, dried, sputter-coated with gold, and examined by field emission scanning electron microscopy (FE-SEM; Guoyi Quantum, Suzhou, China) at 10 kV in backscattered electron mode. Six randomly selected interface images (1,500× magnification) per slab were analyzed using ImageJ software to quantify silver nitrate penetration within the hybrid layer (Tjaderhane et al., 2013).

#### 2.4.3 *In-situ* zymography of the bonding interface

Resin-dentin slabs were prepared as described; however, the adhesive was labeled with 0.1% rhodamine B isothiocyanate. Slabs were polished (600–2000 grit), ultrasonically cleaned, placed onto microscope slides, and coated with 50 μL collagenase activity indicator. Slides were incubated at 37°C in a humidified, dark environment for 48 h, rinsed with deionized water, and dried. Endogenous enzyme activity at the bonding interface was examined by confocal laser scanning microscopy (CLSM; Nikon A1, Nikon Corp., Tokyo, Japan) at 488/530 nm using a ×20 objective lens. Two random images per specimen (n = 6 images per group) were captured, and green fluorescence indicating enzyme activity was quantified using ImageJ software (Gu et al., 2018).

#### 2.4.4 Collagen crosslinking analysis (SDS-PAGE)

Collagen crosslinking was assessed using sodium dodecyl sulfate-polyacrylamide gel electrophoresis (SDS-PAGE). All solutions were stored at room temperature for 24 h before analysis. Experimental Groups: the marker group, non-crosslinked control group (Deionized water: 3.52 mg/mL collagen solution = 1:1), positive control group (5% glutaraldehyde [GD]), and CLM-treated group. Each group was mixed with 10 μL of SDS protein loading buffer, heated at 98°C for 10 min, and 10 μL of each sample was loaded into the gel. Electrophoresis was performed at 80 V for 30 min, followed by 100 V until the marker reached the bottom. After electrophoresis, gels were stained with Coomassie blue for 2 h, destained for 1 h, and analyzed (triplicate for each group) (Wu et al., 2019).

### 2.5 Antibacterial activity

#### 2.5.1 Colony-forming units (CFU)

The antibacterial activity of CLM was evaluated against *S. mutans* (*Streptococcus mutans*; UA159, Yunnan Key Laboratory of Stomatology and Department of Dental Research). *Streptococcus mutans* was cultured anaerobically (85% N_2_, 5% CO_2_, 10% H_2_) at 37°C for 24 h. A bacterial suspension of 1 × 10^6^ CFU/mL was prepared.

CLM was dissolved at 5 mg/mL, mixed in equal proportions with the bacterial suspension, and incubated with shaking for 24 h. One hundred microliters of the 10-fold diluted solution was plated onto brain heart infusion agar plates and cultured for 48 h, after which colony counts were recorded (n = 6) (Daabash et al., 2023).

#### 2.5.2 Biofilm observation

Sterile glass coverslips placed in 6-well plates were inoculated with 1 mL of *S. mutans* suspension and CLM solution, incubated anaerobically at 37°C for 24 h to form biofilms, fixed with 2.5% glutaraldehyde (GD), dehydrated in ethanol gradients (75%–100%), air-dried, gold-sputtered, and examined using FE-SEM at ×20,000 magnification (n = 3) (Akram et al., 2022).

#### 2.5.3 Live/dead bacterial staining

After biofilm formation, each well was treated with 500 μL of the LIVE/DEAD BacLight stain (L7012, Molecular Probes, Thermo Fisher Scientific). The specimens were incubated at room temperature in the dark for 15 min, rinsed with PBS, and observed by CLSM using a ×20 objective lens. Image analyses were performed using ImageJ software (NIH, Bethesda, MD, United States) (Cheng et al., 2012).

### 2.6 Statistical analysis

Data were analyzed using SPSS 20.0 software (IBM Corp., Armonk, NY, United States). The Shapiro–Wilk test and the modified Levene test were used to assess the normality and homogeneity of variance of individual datasets before applying parametric statistical methods. Data from μTBS, nanoleakage assessment and *in-situ* zymography were analyzed using the one-way analysis of variance, followed by the *post hoc* Bonferroni test for multiple comparisons. Data from CFU assays and live/dead bacterial staining were analyzed using independent samples t-tests. For all statistical analyses, the threshold for statistical significance was set at α = 0.05.

## 3 Results

### 3.1 Characterization of CLM-Collagen binding

#### 3.1.1 FTIR characterization

**FIGURE 3:**
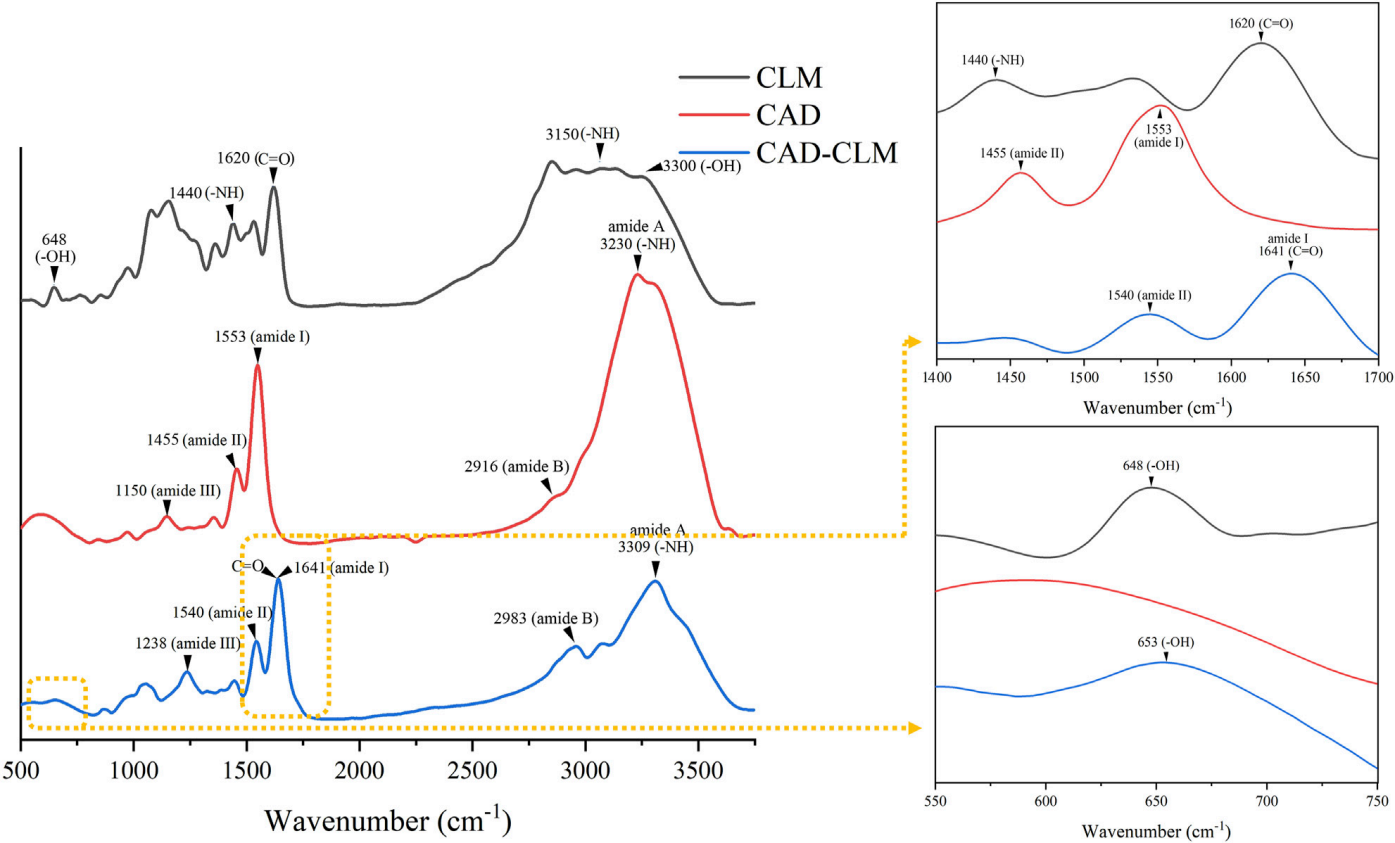
Infrared (FTIR) spectra showing interactions between CAD and CLM. The FTIR analysis demonstrated that CLM effectively bonded with CAD collagen.

As shown in Figure 3, both CLM-treated and untreated CAD exhibited characteristic dentin collagen peaks, including amide A, amide B, amide I, amide II, and amide III. After CLM treatment, a blue shift was observed in the amide I (1,641 cm^−1^), amide II (1,540 cm^−1^), and amide III (1,238 cm^−1^) peaks of CAD. Additionally, the appearance of CLM-specific -OH (653 cm^−1^) and C=O (1,641 cm^−1^) peaks confirmed that CLM was successfully grafted onto the collagen surface.

#### 3.1.2 NMR characterization

**FIGURE 4:**
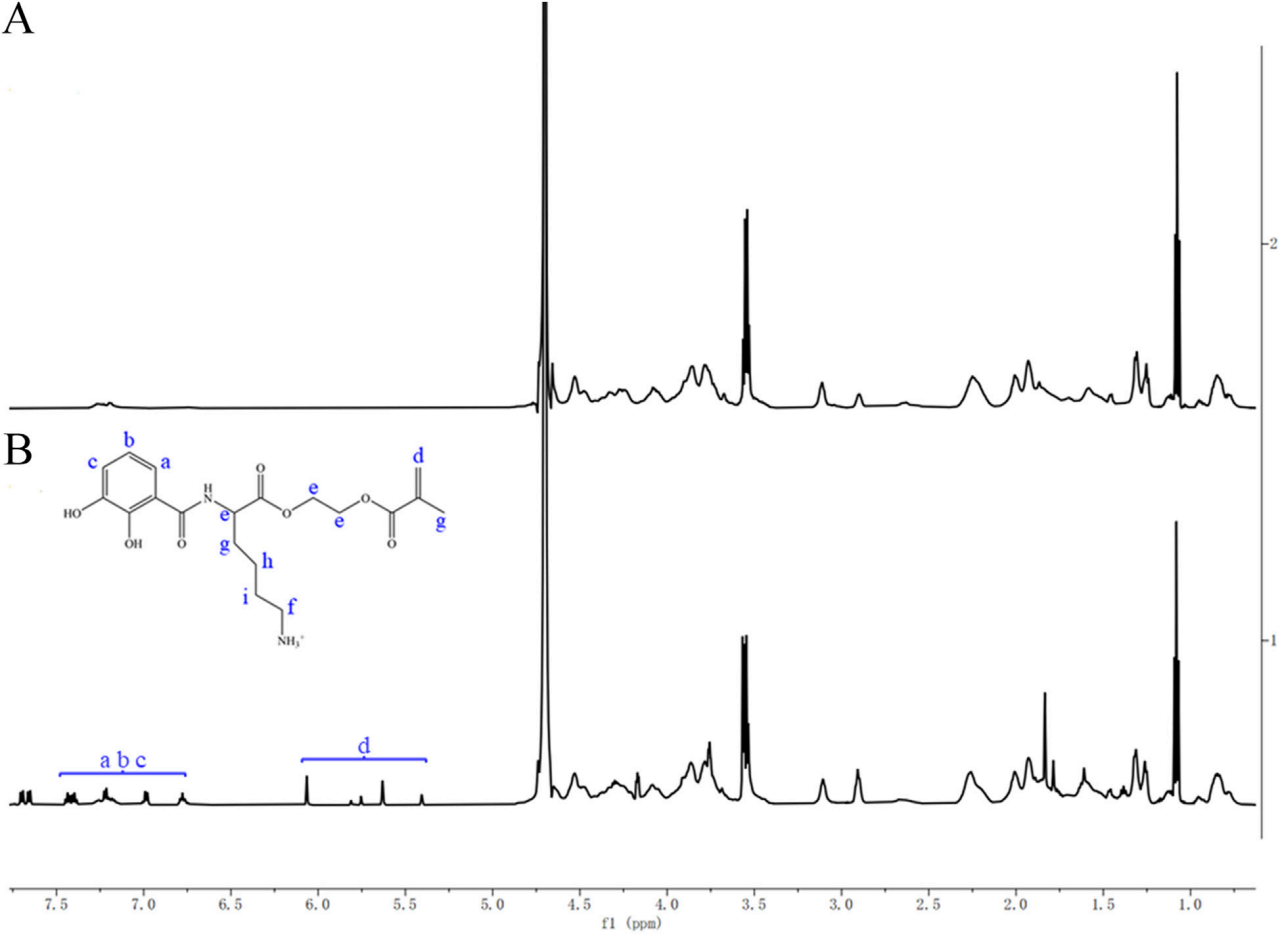
^1^H NMR spectra of Type I collagen before and after interaction with CLM. **(A)** Type I collagen alone. **(B)** Type I collagen after interaction with CLM. NMR spectra showed characteristic peaks of catechol (6.7–7.5 ppm) and methacrylate groups (5.4–6.1 ppm) after interaction with collagen, confirming effective bonding between CLM and Type I collagen.

Figure 4A shows the ^1^H NMR spectrum of Type I collagen, and Figure 4B represents the ^1^H NMR spectrum after reacting Type I collagen with CLM. Compared with collagen alone, the reaction between CLM and collagen resulted in additional peaks: peaks at 6.7–7.5 ppm correspond to hydrogen atoms (a, b, c) on the benzene ring of CLM, and peaks at 5.4–6.1 ppm correspond to hydrogen atoms on the acrylic double bond (d). These findings confirm that CLM successfully bonded with collagen from CAD.

### 3.2 Dentin bonding performance

#### 3.2.1 Microtensile bond strength (µTBS)

**FIGURE 5:**
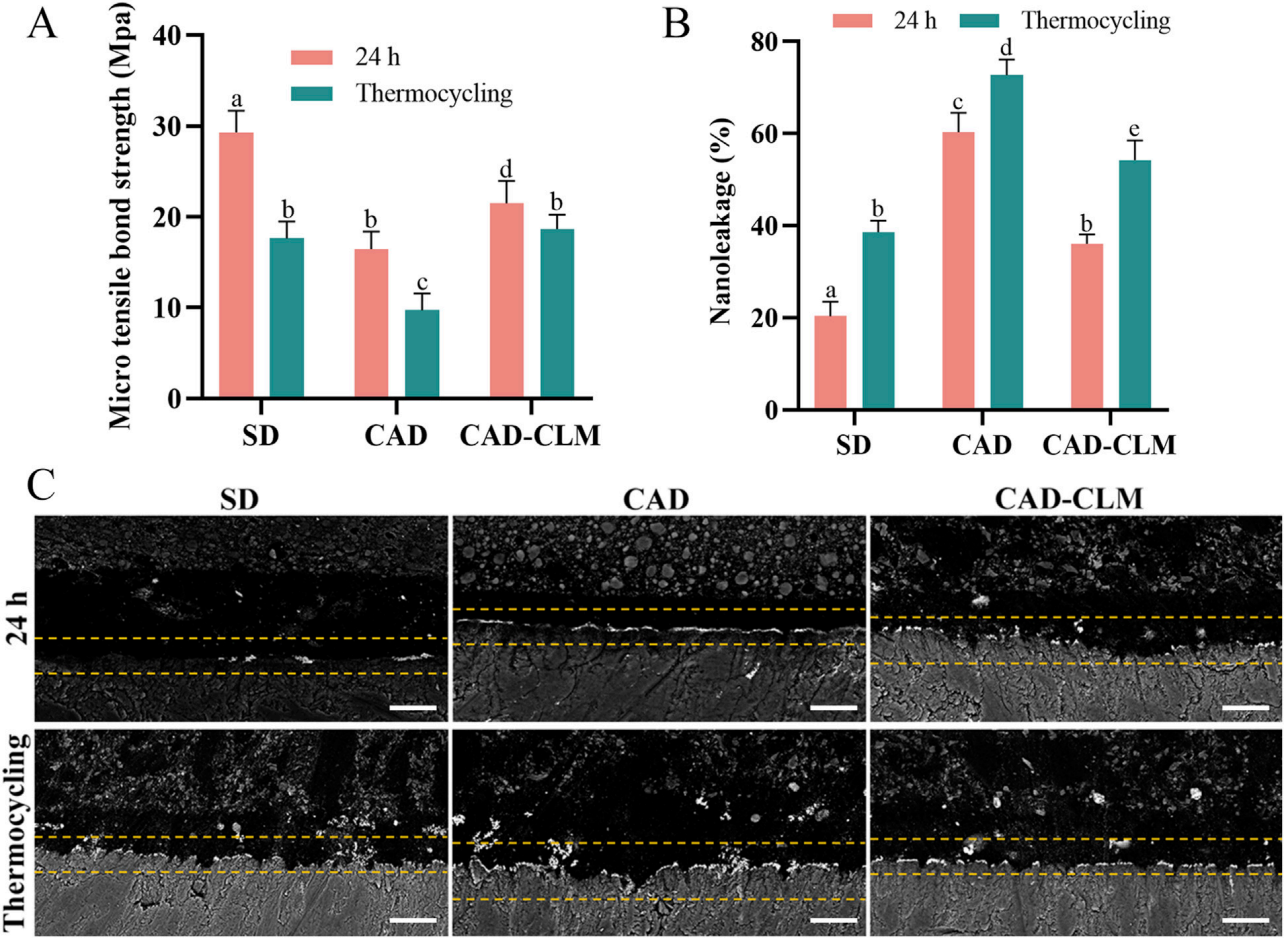
CLM treatment enhances CAD bonding performance. **(A)** Microtensile bond strength (μTBS) results for each group before and after thermocycling. CLM pretreatment significantly improved the strength of immediate and aged bonds to CAD. **(B)** Semiquantitative nanoleakage analyses before and after thermocycling. **(C)** Representative backscattered FE-SEM micrographs showing typical nanoleakage features (1,500×, Bar = 10 μm). Reduced nanoleakage in CLM-treated CAD indicates improved bonding integrity. All data are expressed as the mean ± standard deviation and were analyzed using the one-way ANOVA with *post hoc* Bonferroni tests. Different letters indicate statistically significant differences between groups (p < 0.05).

The strength of the resin-dentin bond is influenced by substrate type and aging conditions. µTBS results at immediate (24 h) and after aging by thermocycling are shown in Figure 5A. Immediately after bonding, the µTBS of the CAD group (16.4 ± 1.9 MPa) was significantly lower (by about 44%) compared to the SD group (29.3 ± 2.4 MPa, p < 0.001). Treatment with the CLM primer significantly improved the µTBS of the CAD-CLM group (21.5 ± 2.5 MPa) compared to the untreated CAD group (p < 0.001). After thermocycling, bond strengths decreased in all groups compared to immediate testing. The SD group decreased by approximately 40% (17.6 ± 1.8 MPa), the CAD group by 41% (9.7 ± 1.8 MPa), while the CAD-CLM group decreased by only about 13% (18.7 ± 1.6 MPa). After aging, the CAD group showed the lowest µTBS (p < 0.001). No significant difference was found between the CAD-CLM group and the aged SD group (p = 1.0).

#### 3.2.2 Nanoleakage evaluation

Nanoleakage was used to evaluate the sealing performance of the bonding interface (Figures 5B,C). Immediately after bonding, the SD group exhibited the lowest nanoleakage (20.4% ± 3.1%, p < 0.001), while the CAD group showed the highest (60.3% ± 4.2%, p < 0.001). The CAD-CLM group (38.5% ± 2.6%) had significantly reduced nanoleakage compared with the CAD group (p < 0.001). After thermocycling, nanoleakage increased in all groups compared with their immediate results (p < 0.001). After aging, the CAD group continued to show the highest nanoleakage (72.7% ± 3.4%, p < 0.001). The aged CAD-CLM group (54.2% ± 4.2%) had significantly lower nanoleakage than the aged CAD group (p < 0.001), similar to the immediate CAD results (p = 0.058).

#### 3.2.3 *In-situ* zymography of the bonding interface

**FIGURE 6:**
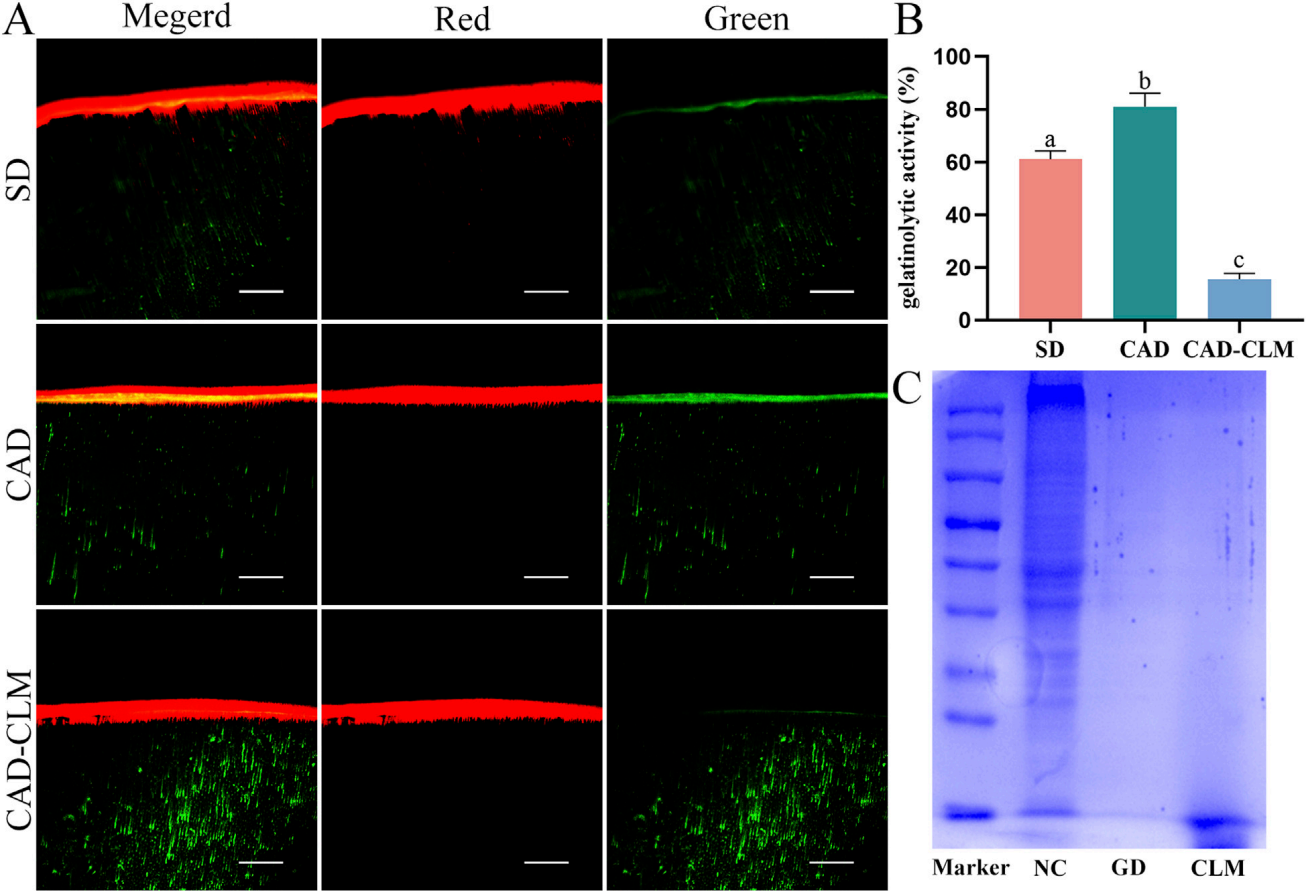
CLM pretreatment reduces the risk of collagen degradation. **(A)** *In situ* zymography showing gelatinolytic activity in the dentin hybrid layer for each subgroup, observed *via* CLSM (20×, Bar = 50 μm). **(B)** Relative percentage of gelatinolytic activity within the hybrid layers. The results indicated that CLM significantly reduced endogenous enzymatic activity, thus mitigating enzymatic degradation risks at the bonding interface. **(C)** SDS-PAGE analysis of collagen modified by cross-linking. The CLM and positive control (GD) groups showed no detectable protein bands, confirming significant collagen cross-linking. *In situ* zymography data are presented as the mean ± standard deviation and were analyzed using the one-way ANOVA with *post hoc* Bonferroni tests. Columns labeled with different letters indicate statistically significant differences (p < 0.05).

In the *in-situ* zymography profiling experiment (Figures 6A,B), the immediate CAD group exhibited the highest endogenous enzyme activity (81.0% ± 5.0%, p < 0.001). In contrast, enzyme activity was significantly lower in the CAD-CLM group (15.6% ± 2.2%) than in the SD (61.0% ± 3.2%) and CAD groups (p < 0.001).

#### 3.2.4 SDS-PAGE collagen crosslinking evaluation

The collagen crosslinking effect of CLM was evaluated by SDS-PAGE (Figure 6C). In the uncross-linked control, multiple distinct collagen protein bands were observed, indicating fragmented collagen. In contrast, no collagen bands appeared in the positive control (GD) and CLM groups, suggesting effective collagen crosslinking in these samples.

### 3.3 Antibacterial performance

**FIGURE 7:**
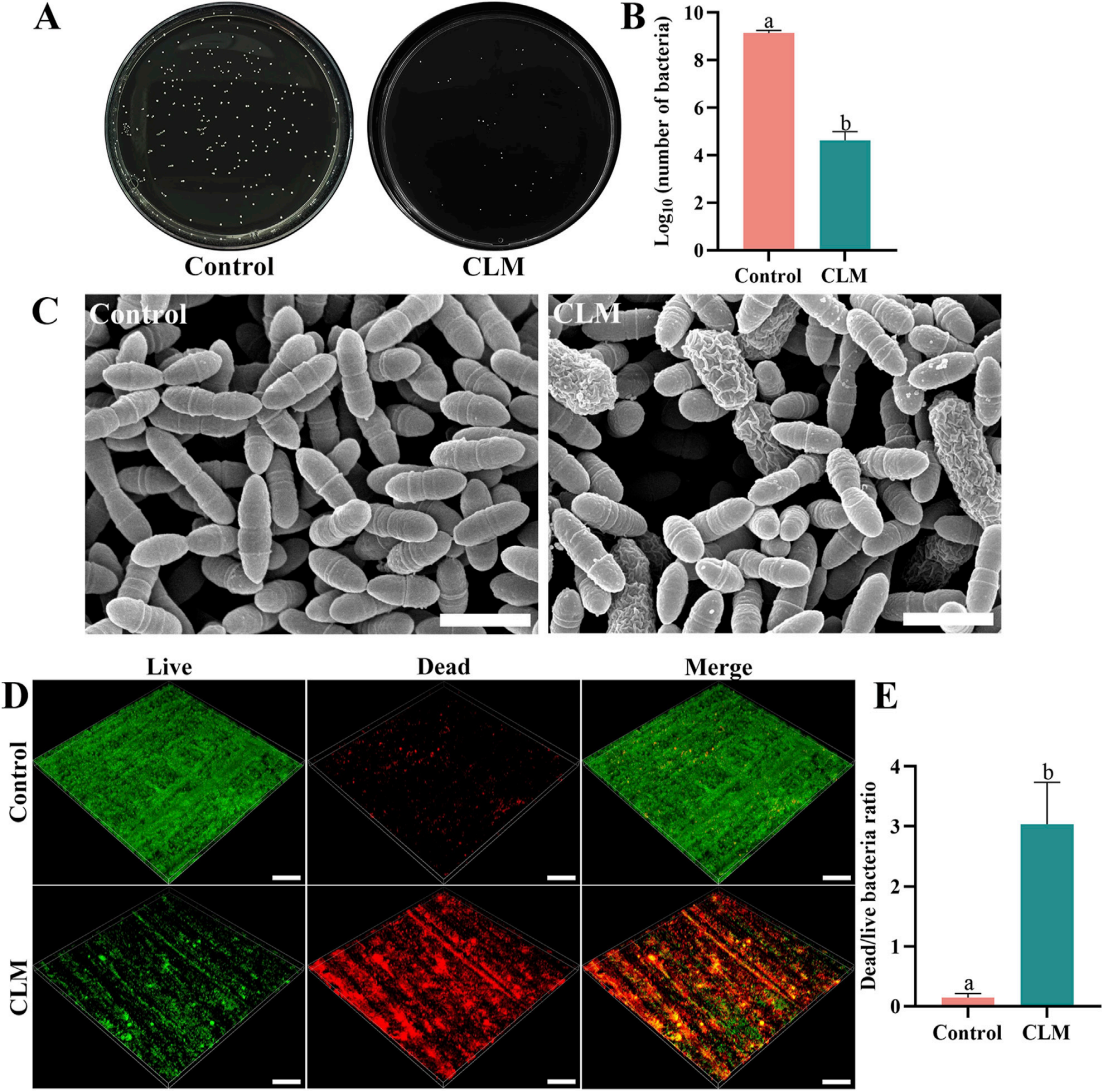
Antibacterial activity of CLM. **(A)** CFU assay on a solid culture medium. **(B)** CFU counts of *Streptococcus mutans* after co-cultivation with CLM. CFU results demonstrated significant bacterial colony growth inhibition by CLM. **(C)** FE-SEM images of biofilms after CLM treatment (20,000×, Bar = 10 μm), showing bacterial cell swelling and membrane shrinkage indicative of cell death. **(D)** Representative 3D reconstructions of *Streptococcus mutans* biofilms (20×, Bar = 200 μm). **(E)** Dead/live bacteria ratio based on live/dead bacterial staining of biofilms. Results showed that CLM reduced overall bacterial numbers and significantly increased the ratio of dead bacteria, confirming strong antibacterial activity. Data in panels B and E are presented as the mean ± standard deviation and were analyzed using independent samples t-tests. Columns labeled with different letters indicate statistically significant differences (p < 0.05).

In the CFU assay (Figures 7A,B), bacterial counts were significantly lower in the CLM group (4.6 ± 0.4) than in the control group (9.1 ± 0.1, p < 0.001). SEM images revealed abundant (Figure 7C), structurally intact bacteria in the control group, while the CLM group exhibited fewer bacteria with noticeable morphological changes indicative of bacterial death, including swelling and membrane shrinkage. Three-dimensional live/dead staining images showed predominantly live bacteria (green) in the control group, whereas the CLM-treated group had predominantly dead bacteria (red). The dead/live bacterial ratio was significantly higher in the CLM group (3.04 ± 0.70) than in the control group (0.15 ± 0.06, p < 0.001; Figures 7D,E). These results confirm that CLM effectively inhibits the growth of *S. mutans*.

## 4 Discussion

Biomodification has emerged as a promising biomimetic approach in dentistry, aiming to enhance the biochemical properties of dental substrates for both preventive and therapeutic applications (Fakhri et al., 2020; Wang et al., 2022). To the best of our knowledge, this study is the first to introduce a mussel-inspired monomer to improve CAD bonding performance. Our results demonstrated that immediate and aged µTBS significantly improved in the CAD-CLM group compared with untreated CAD (p < 0.001), and nanoleakage was significantly reduced (p < 0.001), thus rejecting the first null hypothesis.

In current adhesive systems, self-etch adhesives containing 10-MDP form a stable, low-solubility nanolayer at the bonding interface. The MDP-Ca salt bonding mechanism enhances bond strength and resists degradation, thereby improving dentin bonding durability (Fehrenbach et al., 2021). However, in CAD, reduced mineral density, exposed collagen fibers, and hydroxyapatite loss deprive 10-MDP of bonding sites, limiting its effectiveness. Consequently, etch-and-rinse adhesives achieve better immediate CAD bonding strength than self-etch adhesives (Ceballos et al., 2003). Although CAD has no mineral content, the abundant exposed collagen provides numerous -NH_2_ groups (Matos et al., 2022; Mazzoni et al., 2015), offering favorable targets for CLM chemical bonding. Infrared spectroscopy confirmed the presence of phenolic hydroxyl (-OH, 653 cm^-1^) and ester carbonyl (-C=O, 1,641 cm^−1^) groups on the CAD surface after CLM treatment. Additionally, ^1^H NMR spectra showed peaks at 6.7–7.5 ppm (benzene ring, a, b, c) and 5.4–6.1 ppm (acrylate group, d), confirming successful CLM-collagen binding. Furthermore, the blue shift of amide I–III, amide A and B bands indicated hydrogen bond interactions, while the ester carbonyl (-C=O) signals imply covalent bonding, consistent with previously established reaction mechanisms between CLM and collagen proteins. The lysine residues in the CLM molecule are designed to initially repel bound interfacial water, facilitating more effective contact and subsequent reactions between catechol groups and collagen (Rapp et al., 2016). Moreover, the chemical interaction between CLM and collagen involves a transition from weaker hydrogen bonds to stronger covalent bonds. Initially, catechol groups of CLM form hydrogen bonds with carbonyl or hydroxyl groups on collagen, assisting preliminary adsorption and positioning on the collagen surface. Subsequently, under neutral or slightly alkaline conditions, catechol groups are oxidized into quinones, serving as active electrophilic groups capable of undergoing Michael addition reactions or forming Schiff base linkages with the amino groups (-NH_2_) of collagen, resulting in stable covalent crosslinking (Hu et al., 2023). Thus, initial hydrogen-bonding interactions guide the approach and alignment of molecules, eventually leading to stable covalent cross-links and establishing a strong, durable connection between CLM and collagen structures.

Nanoleakage is widely used to evaluate the quality of the bonding interface (Tjaderhane et al., 2013). In CAD, the loss of minerals and the collapse of collagen networks hinder resin infiltration, resulting in a sparse and porous hybrid layer with more exposed collagen at the interface bottom (Fialho et al., 2019). Therefore, the CAD bonding interface tends to degrade faster than that of SD. According to our nanoleakage results, the immediate leakage rate in the CAD group was three times higher than in the SD group, consistent with the findings of previous studies (Breschi et al., 2018). As an intermediary agent, CLM strongly binds chemically to collagen and introduces methacrylate groups onto the collagen surface, enhancing its hydrophobicity. Moreover, the double bonds (-C=C) in the methacrylate groups likely reside at the periphery of collagen fibers, enabling free radical polymerization with resin monomers during bonding procedures. This promotes resin infiltration and copolymerization. In our previous study, it was demonstrated that 5 mg/mL CLM used as a primer did not significantly affect the polymerization conversion rate of the adhesive resin (Hu et al., 2023). Additionally, the oxidation of catechol groups in CLM typically occurs slowly in the absence of catalysts. Thus, CLM serves as a bridging structure, maintaining collagen integrity and increasing the chemical bond density at the collagen-resin interface, thereby enhancing hybrid layer polymerization and continuity. At low concentrations, CLM does not adversely affect the polymerization of the adhesive resin. Consequently, CLM reduces nanoleakage in CAD, maintaining a more structurally intact bonding interface.

Enzymatic collagen degradation is a primary cause of poor bonding durability. During caries formation, collagen fibers become exposed due to mineral loss, and microbial proteases, host-derived matrix metalloproteinases (MMPs), and cysteine cathepsins become progressively activated, degrading the collagen structure (Isolan et al., 2018; Tang et al., 2023). Thus, enzyme activity is significantly higher in CAD compared to SD, making CAD interfaces more susceptible to enzymatic degradation (Mazzoni et al., 2015; Vidal et al., 2014). In our *in-situ* zymography tests, CAD exhibited the highest endogenous enzyme activity, whereas CLM treatment significantly reduced enzyme activity at the CAD interface. SDS disrupts non-covalent interactions, leaving covalent bonds intact (Chen et al., 2011). In SDS-PAGE analyses, CLM and the positive control (glutaraldehyde, GD) covalently cross-linked collagen, increasing the molecular weight and preventing visible protein bands, thereby confirming CLM’s crosslinking ability.

CLM potentially reduces enzymatic degradation risks in CAD *via* two cooperative mechanisms: 1. Direct inhibition of enzyme activity: Catechol groups in CLM can form covalent bonds with lysine or histidine residues within the active sites of MMPs, effectively blocking catalytic functions. Additionally, catechol groups can chelate metal ions (Zn^2+^ or Ca^2+^), competitively inhibiting the catalytic center of MMPs (Chung et al., 2004). 2. Enhanced collagen stability through crosslinking: CLM-induced hydrogen and covalent bonding between collagen molecules leads to denser collagen structures, making substrate recognition by enzymes more challenging, thus enhancing resistance against enzymatic degradation (Nagase et al., 2008). These dual mechanisms improve collagen stability and mitigate degradation risks, supporting the rejection of the second hypothesis.

Compared to traditional collagen crosslinking agents like GD and proanthocyanidin (PA), CLM offers advantages in biocompatibility and reaction mechanisms. Although GD is an effective cross-linker capable of forming Schiff base cross-links with lysine residues in collagen, consequently improving dentin bonding durability, its ability to enhance the immediate bond strength remains controversial (Al-Ammar et al., 2009). Additionally, GD’s potential cytotoxicity restricts its clinical application (Frassetto et al., 2016). PA, a natural cross-linker with good biocompatibility, stabilizes collagen *via* hydrogen, ionic, and covalent bonds (Vidal et al., 2016). However, PA’s relatively large molecular weight necessitates longer reaction times (10–60 min) (Moreira et al., 2017). By contrast, CLM has dual reactive sites—the catechol group can form covalent bonds with collagen through phenolic condensation reactions, while the methacrylate group provides free radical polymerization points for resin bonding, bridging collagen and resin structures effectively (Hu et al., 2023). Consequently, CLM not only increases collagen crosslinking but also improves resin bonding, combining structural stability with multifunctionality, making it especially promising for complex CAD conditions.

During caries restoration, cariogenic bacteria often remain at the cavity floor and within dentinal tubules. Bacterial proliferation and acid production further activate proteases, increasing the secondary caries risk (Kim et al., 2020). Thus, antibacterial activity within the hybrid layer is essential (Gou et al., 2018). CLM demonstrated antibacterial properties, evaluated using *S. mutans* as a representative cariogenic bacterium. Although a single-species biofilm model is simpler than multi-species biofilms, it ensures consistent preliminary data. CFU counts and live/dead staining results indicated that CLM significantly inhibited *S. mutans* growth, increasing the proportion of dead bacteria. SEM analyses revealed reduced bacterial accumulation and bacteria exhibiting swollen and shriveled morphologies in the CLM group. The antibacterial effect of CLM is attributed to both catechol and lysine groups. Catechol chelates proteins (disrupting bacterial membranes) and generates reactive oxygen species, providing remote antibacterial activity. Positively charged lysine disrupts negatively charged bacterial membranes, delivering contact antibacterial action (Fu et al., 2021; Lan et al., 2021; Liu et al., 2019).

Nevertheless, this study has certain limitations as an *in vitro* investigation. Although μTBS is widely employed for evaluating mechanical properties, it is a static method that does not entirely replicate oral function. Furthermore, the use of a single bacterial species and the absence of long-term biological performance studies represent additional limitations. Future research should include long-term aging studies, comprehensive biocompatibility assessments, and optimal clinical strategy identification for CLM application to support potential clinical utilization.

## 5 Conclusion

The CLM primer effectively leverages the abundant collagen present in CAD, significantly enhancing both immediate and long-term bond strength. In future studies, aimed at deeper understanding of the structural characteristics of CAD—and strategically harnessing the chemical bonding potential of its key components—will offer a promising pathway for further enhancing bonding performance. This remains a central focus of our ongoing research.

## Data Availability

The original contributions presented in the study are included in the article/supplementary material, further inquiries can be directed to the corresponding authors.

## References

[B1] Akram Z. Aati S. Clode P. Saunders M. Ngo H. Fawzy A. S. (2022). Formulation of nano-graphene doped with nano silver modified dentin bonding agents with enhanced interfacial stability and antibiofilm properties. Dent. Mater. 38 (2), 347–362. 10.1016/j.dental.2021.12.016 34930621

[B2] Al-Ammar A. Drummond J. L. Bedran-Russo A. K. (2009). The use of collagen cross-linking agents to enhance dentin bond strength. J. Biomed. Mater. Res. Part B 91 (1), 419–424. 10.1002/jbm.b.31417 PMC2771399 19507140

[B3] Almahdy A. Koller G. Festy F. Bartsch J. W. Watson T. F. Banerjee A. (2015). An mmp-inhibitor modified adhesive primer enhances bond durability to carious dentin. Dent. Mater. 31 (5), 594–602. 10.1016/j.dental.2015.03.003 25804191

[B4] Breschi L. Maravic T. Cunha S. R. Comba A. Cadenaro M. Tjaderhane L. (2018). Dentin bonding systems: from dentin collagen structure to bond preservation and clinical applications. Dent. Mater. 34 (1), 78–96. 10.1016/j.dental.2017.11.005 29179971

[B5] Cadenaro M. Josic U. Maravic T. Mazzitelli C. Marchesi G. Mancuso E. (2023). Progress in dental adhesive materials. J. Dent. Res. 102 (3), 254–262. 10.1177/00220345221145673 36694473

[B6] Ceballos L. Camejo D. G. Victoria F. M. Osorio R. Toledano M. Carvalho R. M. (2003). Microtensile bond strength of total-etch and self-etching adhesives to caries-affected dentine. J. Dent. 31 (7), 469–477. 10.1016/s0300-5712(03)00088-5 12927458

[B7] Chen R. Wang J. B. Zhang X. Q. Ren J. Zeng C. M. (2011). Green tea polyphenol epigallocatechin-3-gallate (egcg) induced intermolecular cross-linking of membrane proteins. Arch. Biochem. Biophys. 507 (2), 343–349. 10.1016/j.abb.2010.12.033 21211509

[B8] Cheng L. Weir M. D. Xu H. H. Antonucci J. M. Kraigsley A. M. Lin N. J. (2012). Antibacterial amorphous calcium phosphate nanocomposites with a quaternary ammonium dimethacrylate and silver nanoparticles. Dent. Mater. 28 (5), 561–572. 10.1016/j.dental.2012.01.005 22305716 PMC3322309

[B9] Chung L. Dinakarpandian D. Yoshida N. Lauer-Fields J. L. Fields G. B. Visse R. (2004). Collagenase unwinds triple-helical collagen prior to peptide bond hydrolysis. EMBO. J. 23 (15), 3020–3030. 10.1038/sj.emboj.7600318 15257288 PMC514933

[B10] Daabash R. Alqahtani M. Q. Price R. B. Alshabib A. Niazy A. Alshaafi M. M. (2023). Surface properties and streptococcus mutans biofilm adhesion of ion-releasing resin-based composite materials. J. Dent. 134, 104549. 10.1016/j.jdent.2023.104549 37196686

[B11] Davila-Sanchez A. Gutierrez M. F. Bermudez J. P. Mendez-Bauer M. L. Hilgemberg B. Sauro S. (2020). Influence of flavonoids on long-term bonding stability on caries-affected dentin. Dent. Mater. 36 (9), 1151–1160. 10.1016/j.dental.2020.05.007 32620332

[B12] Donmez N. Gungor A. S. Karabulut B. Siso S. H. (2019). Comparison of the micro-tensile bond strengths of four different universal adhesives to caries-affected dentin after er:yag laser irradiation. Dent. Mater. J. 38 (2), 218–225. 10.4012/dmj.2017-428 30504693

[B13] Fakhri E. Eslami H. Maroufi P. Pakdel F. Taghizadeh S. Ganbarov K. (2020). Chitosan biomaterials application in dentistry. Int. J. Biol. Macromol. 162, 956–974. 10.1016/j.ijbiomac.2020.06.211 32599234

[B14] Fehrenbach J. Isolan C. P. Munchow E. A. (2021). Is the presence of 10-mdp associated to higher bonding performance for self-etching adhesive systems? A meta-analysis of *in vitro* studies. Dent. Mater. 37 (10), 1463–1485. 10.1016/j.dental.2021.08.014 34456050

[B15] Fialho M. Hass V. Nogueira R. P. Franca F. Turssi C. P. Basting R. T. (2019). Effect of epigallocatechin-3- gallate solutions on bond durability at the adhesive interface in caries-affected dentin. J. Mech. Behav. Biomed. Mater. 91, 398–405. 10.1016/j.jmbbm.2018.11.022 30669058

[B16] Frassetto A. Breschi L. Turco G. Marchesi G. Di Lenarda R. Tay F. R. (2016). Mechanisms of degradation of the hybrid layer in adhesive dentistry and therapeutic agents to improve bond durability--a literature review. Dent. Mater. 32 (2), e41–e53. 10.1016/j.dental.2015.11.007 26743967

[B17] Fu Y. Yang L. Zhang J. Hu J. Duan G. Liu X. (2021). Polydopamine antibacterial materials. Mater. Horiz. 8 (6), 1618–1633. 10.1039/d0mh01985b 34846495

[B18] Gou Y. P. Meghil M. M. Pucci C. R. Breschi L. Pashley D. H. Cutler C. W. (2018). Optimizing resin-dentin bond stability using a bioactive adhesive with concomitant antibacterial properties and anti-proteolytic activities. Acta Biomater. 75, 171–182. 10.1016/j.actbio.2018.06.008 29883811

[B19] Gu L. Mazzoni A. Gou Y. Pucci C. Breschi L. Pashley D. H. (2018). Zymography of hybrid layers created using extrafibrillar demineralization. J. Dent. Res. 97 (4), 409–415. 10.1177/0022034517747264 29294298

[B20] Hass V. Da M. O. T. Cardenas A. de Siqueira F. Bauer J. R. Abuna G. (2021). Is it possible for a simultaneous biomodification during acid etching on naturally caries-affected dentin bonding? Clin. Oral Investig. 25 (6), 3543–3553. 10.1007/s00784-020-03677-8 33200282

[B21] Hiraishi N. Tochio N. Kigawa T. Otsuki M. Tagami J. (2013). Monomer-collagen interactions studied by saturation transfer difference nmr. J. Dent. Res. 92 (3), 284–288. 10.1177/0022034512474310 23340212

[B22] Hu Z. Wu W. Yu M. Wang Z. Yang Z. Xing X. (2023). Mussel-inspired polymer with catechol and cationic lys functionalities for dentin wet bonding. Mater. Today bio. 18, 100506. 10.1016/j.mtbio.2022.100506 PMC9719097 36471892

[B23] Isolan C. P. Sarkis-Onofre R. Lima G. S. Moraes R. R. (2018). Bonding to sound and caries-affected dentin: a systematic review and meta-analysis. J. Adhes. Dent. 20 (1), 7–18. 10.3290/j.jad.a39775 29399679

[B24] Jain N. Dutt U. Radenkov I. Jain S. (2024). Who's global oral health status report 2022: actions, discussion and implementation. Oral Dis. 30 (2), 73–79. 10.1111/odi.14516 36680388

[B25] Kim D. Barraza J. P. Arthur R. A. Hara A. Lewis K. Liu Y. (2020). Spatial mapping of polymicrobial communities reveals a precise biogeography associated with human dental caries. Proc. Natl. Acad. Sci. U. S. A. 117 (22), 12375–12386. 10.1073/pnas.1919099117 32424080 PMC7275741

[B26] Lan X. Liu Y. Wang Y. Tian F. Miao X. Wang H. (2021). Coaxial electrospun pva/pcl nanofibers with dual release of tea polyphenols and epsilon-poly (l-lysine) as antioxidant and antibacterial wound dressing materials. Int. J. Pharm. 601, 120525. 10.1016/j.ijpharm.2021.120525 33781878

[B27] Li K. Yao C. Sun Y. Wang K. Wang X. Wang Z. (2021). Enhancing resin-dentin bond durability using a novel mussel-inspired monomer. Mater. Today bio. 12, 100174. 10.1016/j.mtbio.2021.100174 PMC8640517 34901824

[B28] Li Y. Cheng J. Delparastan P. Wang H. Sigg S. J. DeFrates K. G. (2020). Molecular design principles of lysine-dopa wet adhesion. Nat. Commun. 11 (1), 3895. 10.1038/s41467-020-17597-4 32753588 PMC7403305

[B29] Liu H. Qu X. Tan H. Song J. Lei M. Kim E. (2019). Role of polydopamine's redox-activity on its pro-oxidant, radical-scavenging, and antimicrobial activities. Acta Biomater. 88, 181–196. 10.1016/j.actbio.2019.02.032 30818052

[B30] Macedo G. V. Yamauchi M. Bedran-Russo A. K. (2009). Effects of chemical cross-linkers on caries-affected dentin bonding. J. Dent. Res. 88 (12), 1096–1100. 10.1177/0022034509351001 19892915 PMC3318032

[B31] Maier G. P. Rapp M. V. Waite J. H. Israelachvili J. N. Butler A. (2015). Adaptive synergy between catechol and lysine promotes wet adhesion by surface salt displacement. Science 349 (6248), 628–632. 10.1126/science.aab0556 26250681

[B32] Matos A. B. Reis M. Alania Y. Wu C. D. Li W. Bedran-Russo A. K. (2022). Comparison of collagen features of distinct types of caries-affected dentin. J. Dent. 127, 104310. 10.1016/j.jdent.2022.104310 36167234

[B33] Mazzoni A. Tjaderhane L. Checchi V. Di Lenarda R. Salo T. Tay F. R. (2015). Role of dentin mmps in caries progression and bond stability. J. Dent. Res. 94 (2), 241–251. 10.1177/0022034514562833 25535202 PMC4300303

[B34] Mohammed H. A. Ali G. A. Baroudi K. (2014). The effect of different disinfecting agents on bond strength of resin composites. Int. J. Dent. 2014, 231235. 10.1155/2014/231235 25477961 PMC4247941

[B35] Moreira M. A. Souza N. O. Sousa R. S. Freitas D. Q. Lemos M. V. De Paula D. M. (2017). Efficacy of new natural biomodification agents from anacardiaceae extracts on dentin collagen cross-linking. Dent. Mater. 33 (10), 1103–1109. 10.1016/j.dental.2017.07.003 28751073

[B36] Nagase H. Fushimi K. (2008). Elucidating the function of non-catalytic domains of collagenases and aggrecanases. Connect. Tissue. Res. 49 (3), 169–174. 10.1080/03008200802151698 18661336

[B37] Rapp M. V. Maier G. P. Dobbs H. A. Higdon N. J. Waite J. H. Butler A. (2016). Defining the catechol-cation synergy for enhanced wet adhesion to mineral surfaces. J. Am. Chem. Soc. 138 (29), 9013–9016. 10.1021/jacs.6b03453 27415839

[B40] Schwendicke F. Frencken J. E. Bjorndal L. Maltz M. Manton D. J. Ricketts D. (2016). Managing carious lesions: consensus recommendations on carious tissue removal. Adv. Dent. Res. 28 (2), 58–67. 10.1177/0022034516639271 27099358

[B41] Suppa P. Ruggeri A. J. Tay F. R. Prati C. Biasotto M. Falconi M. (2006). Reduced antigenicity of type i collagen and proteoglycans in sclerotic dentin. J. Dent. Res. 85 (2), 133–137. 10.1177/154405910608500204 16434730 PMC2245799

[B42] Tang K. Wang F. Dai S. Q. Yang Z. Y. Duan L. Y. Luo M. L. (2023). Enhanced bonding to caries-affected dentin using an isocyanate-based primer. J. Dent. Res. 102 (13), 1444–1451. 10.1177/00220345231199416 37950512

[B43] Tezvergil-Mutluay A. Agee K. A. Hoshika T. Tay F. R. Pashley D. H. (2010). The inhibitory effect of polyvinylphosphonic acid on functional matrix metalloproteinase activities in human demineralized dentin. Acta Biomater. 6 (10), 4136–4142. 10.1016/j.actbio.2010.05.017 20580949 PMC2930042

[B44] Tjaderhane L. Mehtala P. Scaffa P. Vidal C. Paakkonen V. Breschi L. (2013). The effect of dimethyl sulfoxide (dmso) on dentin bonding and nanoleakage of etch-and-rinse adhesives. Dent. Mater. 29 (10), 1055–1062. 10.1016/j.dental.2013.07.014 23942144

[B45] Vidal C. M. Tjaderhane L. Scaffa P. M. Tersariol I. L. Pashley D. Nader H. B. (2014). Abundance of mmps and cysteine cathepsins in caries-affected dentin. J. Dent. Res. 93 (3), 269–274. 10.1177/0022034513516979 24356440

[B46] Vidal C. M. Zhu W. Manohar S. Aydin B. Keiderling T. A. Messersmith P. B. (2016). Collagen-collagen interactions mediated by plant-derived proanthocyanidins: a spectroscopic and atomic force microscopy study. Acta Biomater. 41, 110–118. 10.1016/j.actbio.2016.05.026 27208639 PMC4983101

[B47] Wang Z. Li B. Cai Q. Li X. Yin Z. Li B. (2022). Advances and prospects in antibacterial-osteogenic multifunctional dental implant surface. Front. Bioeng. Biotechnol. 10, 921338. 10.3389/fbioe.2022.921338 35685091 PMC9171039

[B48] Wu L. Shao H. Fang Z. Zhao Y. Cao C. Y. Li Q. (2019). Mechanism and effects of polyphenol derivatives for modifying collagen. ACS Biomater. Sci. Eng. 5 (9), 4272–4284. 10.1021/acsbiomaterials.9b00593 33417783

[B49] Xu L. Neoh K. Kang E. (2018). Natural polyphenols as versatile platforms for material engineering and surface functionalization. Prog. Polym. Sci. 87, 165–196. 10.1016/j.progpolymsci.2018.08.005

[B50] Xu Y. Guan J. Wang Q. Xue R. He Z. Lu X. (2023). Mussel-inspired caries management strategy: constructing a tribioactive tooth surface with remineralization, antibiofilm, and anti-inflammation activity. ACS Appl. Mater. Interfaces 15 (12), 15946–15964. 10.1021/acsami.2c21672 36940092

